# The ethics of performance care: A pragmatic feminist analysis of policy for singing voice rehabilitation

**DOI:** 10.1111/jep.14107

**Published:** 2024-09-30

**Authors:** Jenna Brown

**Affiliations:** ^1^ Voice Study Centre UK University of Wales Trinity St David Bristol UK; ^2^ Present address: Institute of Education, University College London London UK

**Keywords:** ethics, feminism, intersectionality, policy, voice rehabilitation

## Abstract

**Introduction:**

This paper uses pragmatic feminist poststructuralism to explore how ethical theory is applied to singing voice rehabilitation by specialist singing teachers.

**Methods:**

A critical literature review examines the relationship between traditional and feminist ethical theories and their potential impact on practice. Themes have been extracted from the literature to create an intersectional feminist poststructural analysis framework, facilitating a document analysis of the foundations of three policy documents currently available to singing voice rehabilitation specialists. Poststructural deconstructivism was applied to thematic analysis to consider the impact of ethical theories on policy and practice.

**Findings:**

Policies we found to be rooted in traditional enlightenment ethics, with a focus on hyper‐rationality, androcentrism and legalism. Person‐centred care ethics was found to be lacking in all documents. Contrary to best‐practice recommendations documents failed to provided practical guidelines for practitioners.

**Conclusion:**

Findings indicate adopting an intersectional feminist ethical policy could improve existing documents via a move from legislation and authority towards care and reflexivity.

## 1 INTRODUCTION

Singing voice rehabilitation specialists (SVRS) are singing teachers with ‘…extra training to prepare them for work with injured voices, in collaboration with a medical voice team’.^1^ ^,p.115^ Standing at the intersection between pedagogy and clinical practice, SVRSs draw on a range of disciplines, including physiology, psychology and others,^2^, ^3^, ^4^ and regularly meet ethical dilemmas as they navigate interdisciplinary boundaries.^5^, ^6^, ^7^ It is debated whether rehabilitation should only be carried out by registered clinicians^8^ however, it can be suggested singing teachers have a unique position of relational depth,^9^, ^10^ allowing them to gain detailed understanding of client's experiences. Additionally, first‐hand experience as singers strengthens their ability to interpret clients' feedback. This supports best practice requirements that practitioners have extensive performance experience and/or training in voice pedagogy.^11^, ^12^, ^13^ In many clinical contexts this level of training and relational depth is not achievable,^14^, ^15^, ^16^, ^17^ however SVRSs can bridge this gap. Despite this they are not medical professionals^18^ and cannot diagnose, treat (without clinical guidance) or, in psychogenic cases, offer psychotherapy.^19^ Although codes of conduct and scope of practice documents are available to guide practitioners, their utility is questionable. This is in part due to their brevity and also their limited ethical scope, raising questions surrounding the prima facie principles upon which they are based. Who has decided what is ethical? What has been understood as ‘good practice’? What are the structural roots of these documents? What are the biases contained within them?

### 1.1 Systemic context

Contextual analysis suggests that the three core disciplines underpinning SVRS practice (ethics, medicine, and pedagogy) are founded on discriminatory structures.^20^, ^21^ This contention has developed from feminist analysis of enlightenment thinking, which remains the bedrock of current evidence‐based practice.^22^ Within these traditions, there exists a hierarchy of dualisms^23^, ^24^ that prioritises knowledge derived from rational, autonomous thought over that which is gained from relational experience.^25^, ^26^ Critics of such perspectives argue this presents a two‐dimensional understanding of what it is to be human,^27^ which limits the efficacy of methods employed within these disciplines as it undervalues and delegitimises experiences and perceptions that fall outside of enlightenment structures.^28^, ^29^

Extrapolating further to consider the paradigmatic contexts within which SVRS' work, one can evidence a dichotomous relationship between the biopsychosocial (BPS) model that underpins some areas of practice^30^, ^31^ and the structural foundations of evidence‐based western medicine.^32^ It has been argued that structuralist epistemology appears in opposition to more pragmatic person‐centred BPS models,^18^, ^33^ with Western health care founded on patriarchal principles,^34^, ^35^, ^36^ which elevate and prioritise empirical knowledge and interpretation by medical experts over client and practitioner lived experience.^37^, ^38^, ^39^ Aside from challenges raised by this model in regard to valuing patient perception, the structuralist epistemology raises questions about knowledge ‘gatekeeping’ in health care, similar to those found in academia.^40^, ^41^ If patriarchal structuralism is embedded within the ethics that underpin SVRS guidelines, could the bias inherent within that be unfairly restricting scope of practice and client outcomes? What might happen in practice if we deconstruct the antecedents of ethical policy and develop an intersectional feminist SVRS framework and how might this impact the broader pedagogical and clinical communities?

## 2 METHODOLOGY

Pragmatic Feminism,^25^ and feminist poststructuralism^42^ have been synthesised to offer a critical paradigm that examines hierarchical dichotomies through direct challenge of traditional patriarchal structures as they are evidenced in text. Acknowledging the performative role of texts in shaping practice^43^ feminists have harnessed the post‐structural rejection of grand narratives,^44^, ^45^ challenging social structures that rely on absolute and universal truths to legitimise forms of political activity.^42^, ^45^ This addresses bias by casting a critical lens over that which we take as commonplace.^46^ It prompts us to consider how things might be different and how we can create the conditions for growth and development, freeing us from historical structures that no longer represent the best interests of a diverse, multicultural society.

As the documents selected for analysis are grounded in praxis involving people with diverse identities and experiences, a further intersectional lens was applied. A development of feminist theorising^47^ intersectionality offers criticality through which the relationality of practice can be examined.^48^ The Enmarginalised Feminist Policy Analysis Framework^49^ was adapted as a form of epistemological resistance,^48^ to consider the impact of SVRS policy on marginalised social groups.

### 2.1 Methods

The aim of this study is to investigate the ethical foundations of SVRS policy. It seeks answer to the following research questions:

- What are the ethical antecedents of current SVRS ethical policy?
- What are the implications of current SVRS ethical policy documents on SVRS practice when viewed through a pragmatic feminist intersectional and post‐structural lens?
- How might a pragmatic feminist intersectional and post‐structural paradigm contribute to the future development of SVRS ethical policy and practice?

These objectives were realised through the completion of document analysis (DA) achieved using a range of methods inspired by O'Leary's^50^ guidelines for DA.

The documents selected for analysis are the only three currently available specifically for SVRS: British Association of Performance Arts Medicine (BAPAM) VRS Competencies,^51^ Pan American Voice Association (PAVA)^52^ and, Vocal Health Education.^53^ All three are online open access.

#### 2.1.1 Developing an analysis framework

Poststructuralism is concerned with the performative and symbolic nature of language,^54^, ^55^ with texts viewed as not merely representative but also constructive. Therefore, their meaning can be contingent upon both their social‐interpretative context *and* the theories, values, biases and intentions of authors.^56^, ^57^ To uncover these foundations, the post‐structuralist method of deconstruction^58^ has been applied to develop an analysis framework. Derrida^58^ maintained the underlying structures of texts need examination through a double deconstructive reading, to exemplify intended and unintended meanings.^59^ The first reading (identifying explicit intentions) is destabilised by the second, which is executed via a framework of themes and content that is implicit or missing from the traditional reading. In this paper, creation of this double‐deconstructive framework will be informed by best‐practice intersectional feminist policy guidelines and thematic analysis of traditional and feminist ethics (FE) derived from a literature review.

#### 2.1.2 Literature review methodology

Lindemann states that ‘if you don't know how things are, your prescriptions for how things ought to be won't have much practical effect’,^29^,p.17 therefore, developing an understanding of how arguments within ethics have developed over time is essential for understanding the current context. To this end, a critical narrative literature review^60^, ^61^, ^62^ was undertaken to provide contextual information on theories within FE, providing strong theoretical grounding, introducing the field^63^, ^64^ and informing the DA framework.

##### Search strategies

The literature review examines pivotal philosophical texts^60^:

- Utilitarianism^65^, ^66^
- Deontology^67^, ^68^, ^69^
- Social Contract Theory^70^
- Virtue Ethics^71^

These were selected purposively^72^ based on their central position in normative ethics.^29^ The selection criteria for FE warranted more careful consideration than simply selecting pivotal texts, as the definition of these is not so clearcut. The number of feminist studies is vast and beyond the scope of this paper to be considered exhaustively. Therefore, literature will only be reviewed if published between 2017 and 2022, corresponding to SVRS policy documents. Furthermore, aligning publication dates with policy provides clearer insights into the socio‐political‐ethical context within which SVRSs practice.^73^

**Figure 1:**
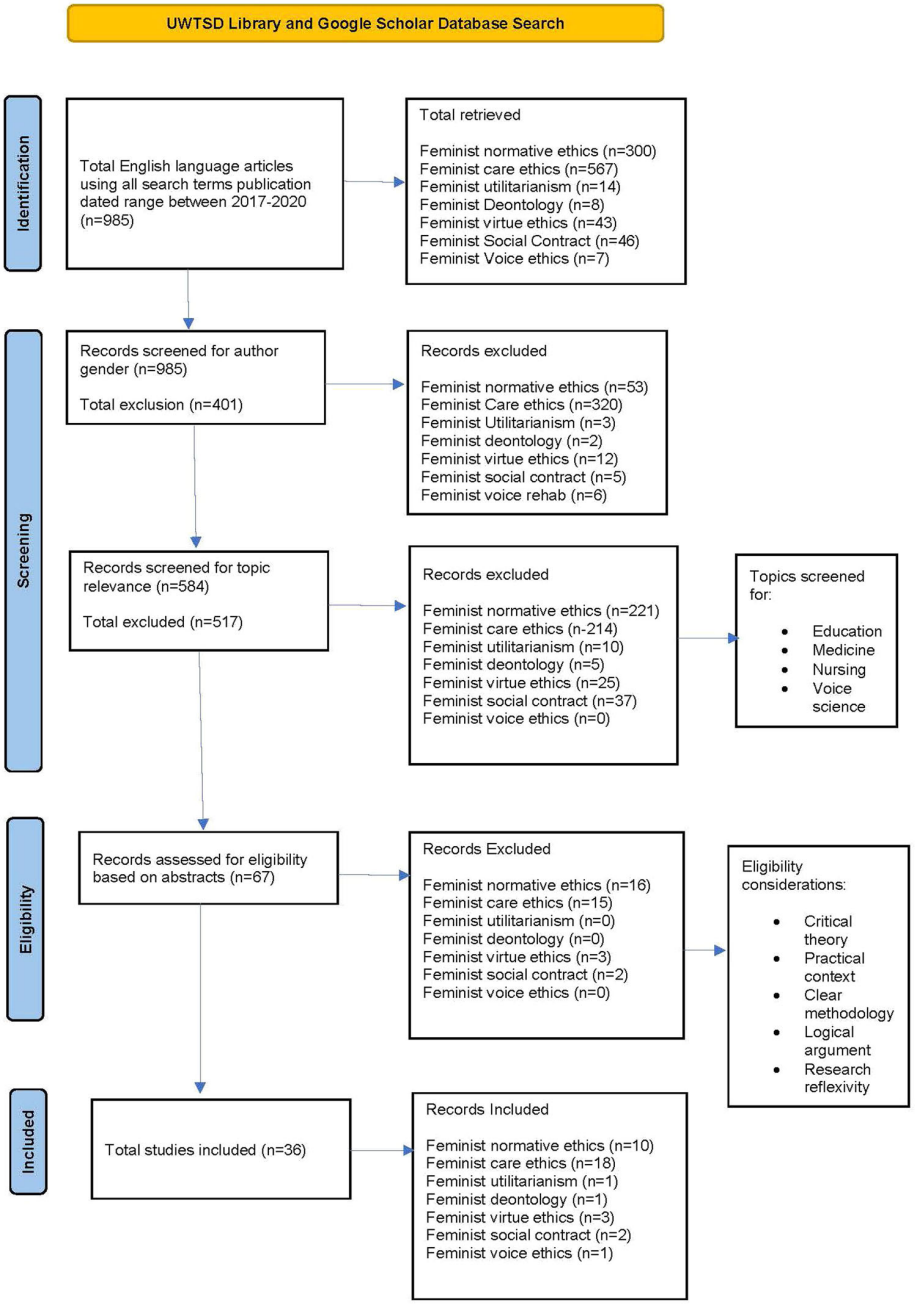
Preferred reporting items for systematic reviews and meta analyses (PRISMA) search criteria.

Traditionally, peer‐reviewed, quantitative studies have been prioritised,^74^ however, this has greatly prejudiced the academic canon in favour of individuals who have more access to opportunities. In line with the values of intersectional feminism, searching beyond orthodoxy, at the margins^75^, ^76^, ^77^ included all types of literature, avoiding hierarchical bias, and supporting the themes of pragmatic feminist post‐structuralism. Femaleauthors were prioritised to redress gender bias and ensure findings emancipate the voices of those traditionally marginalised in academia. Pizzingrilli^78^ highlights the issue of ‘whitewashing’ in feminist research and therefore intersectional analyses was also prioritised (Figure 1). This involved actively searching for literature by non‐White authors, as well as assessing the suitability of material for inclusion using questions from The Enmarginalised Feminist Policy Analysis Framework.^49^

##### Coding and analysis

**Figure 2:**
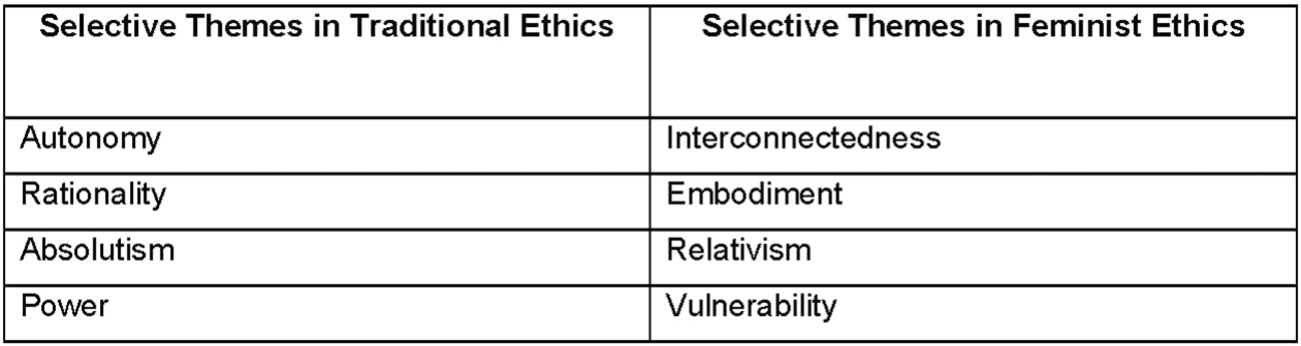
Themes in traditional and feminist ethics.

The documents were then analysed deductively using open, axial and selective coding,^79^, ^80^, ^81^, ^82^ from which core themes in traditional and FE were extracted for use in the DA framework (Figure 2).

The two thematic categories provide a double reading in accordance with deconstructivism,^54^, ^77^, ^83^ addressing the possibility of skewed interpretation by actively seeking contrary viewpoints.^64^, ^84^, ^85^

#### 2.1.3 DA methods

The DA sought to uncover the ethical foundations of the SVRS documents, and therefore the presence of both traditional and feminist ethical themes was investigated. This was achieved through application of O'Leary's ‘Occurrence’ technique^50^ and Bowen's thematic technique.^86^ These were both employed, as combining quantitative and qualitative methods provides a broad base for understanding policy content and context.^86^ Quantitatively, occurrence technique elicited the number of times traditional and feminist themes were present. These were collated using in vivo coding,^87^ providing direct quotations to support findings discussion. Additionally, analysis of language considers the extent to which policy addresses the practical context of SVRS.

Alongside this quantitative approach, O'Leary's ‘Interview’ technique^50^ asks qualitative questions of documents to elicit more in‐depth understanding of how they were produced.^88^ Theoretical and best‐practice literature provide guidance on questions to be asked,^86^ including guidelines from globally recognised leaders in policymaking, Deloitte^89^ and the Chartered Institute of Personnel Development.^90^ Additionally, policies from the British Association of Counselling and Psychotherapy,^52^ The International Coaching Federation,^91^ Health and Care Professionals Council,^92^ and The Teaching Standards Agency^93^ were consulted as organisations recognised as leaders in fields closely aligned with SVRS practice. From a specifically feminist perspective, The Enmarginalised Feminist Policy Analysis Framework^49^ was consulted as a tool which considers intersectionality and researcher positionality, allowing critical investigation of multiple socio‐political systems and multidisciplinary fields such as SVRS. Questions were designed based on each of these frameworks and then ‘asked’ of each policy, eliciting information about their creation and intended outcomes.

## 3 LITERATURE REVIEW

To thoroughly engage with themes in FE it is crucial to understand the traditional theories they critique.^29^ For readers unfamiliar with traditional normative ethics, a summary of theories reviewed is presented below:

1. Social Contract Theory^70^: social cooperation is necessary to prevent anarchy. Cooperation requires development of rules conveying contractual guarantees. Core guarantees are:

a. you will not be harmed
b. others will stick to this agreement

This conditional ethics is concerned with public life and the state is responsible for upholding guarantees through law. Legal content is decided by logical reasoning and rational argument. Key themes are autonomy, rationality and regulation.

2. Deontology (Kant, Critique of Pure Reason date, 1764/2004): ethical behaviour is dutiful. It is not concerned with consequences but whether action is rational and autonomous. There are three guiding maxims:

(1) act only in a way that you would wish it to become universal law
(2) act in a way that does not treat people as means to end
(3) all laws should be harmonised with a kingdom of ends (a world of rational beings who morally legislate based on objective, a priori laws).

Key themes are autonomy, rationality and universality.

3. Utilitarianism (Bentham, Principles of Morals and Legislation, 1780/2007^65^ and Mill, Utilitarianism, 1861/2015)^66^: moral behaviour elicits the greatest happiness for the greatest number. The hedonic calculus is a formula for ethical decision making:

(a) State proposed action
(b) Total happiness—total pain
(c) Number of beneficiaries—number harmed

If the two parts of the calculus agree, you ought to follow the proposed course of action.

Happiness is considered impartially and no context is considered. Key themes are pleasure, universality, rationality and impartiality.

4. Virtue Ethics^71^: ethical action is that which is most virtuous. Behaviour is evaluated against standards of virtue, deficiency and excess. Key themes are reason, logic, autonomy, universality.

### 3.1 FE

Ethics considers how we live^94^ and is often understood in terms of dichotomies: right vs wrong, absolute vs relative, emotive versus rational.^23^ However, FE consideration of socio‐political contexts raises questions about the way we accept traditional moral arguments.^28^ FE argues injustices experienced are resultant of outdated, patriarchal structuralism that underpins traditional ethics (TE).^28^ Furthermore, intersectional feminists argue that eurocentrism and western colonialism have cemented racial hierarchies in social institutions,^95^ which limit ethical practice and reduces some members of society to the margins of care. FE views such sociocultural antecedents of ethical normativity as incongruent with lived experience, with intersectional feminists highlighting this as of particular concern for global majority populations.^76^ Feminists propose a radical shift away from andro‐ and eurocentric ideals of universal rational autonomy to create a fairer society.^28^

#### 3.1.1 Absolutism and relativism

FE is inherently pragmatic,^96^, ^97^ and a major criticism of TE is that absolutism relies on ideals.^98^ Universalising principles state ethical standards should be applied impartially across all situations; however, this fails to recognise human nature as fallible, with conscious and unconscious bias being called out by intersectional scholars such as Tuhiwai Smith,^76^ as a problem for such thought. Nor does it give consideration of context and the need for relativism.^96^, ^98^ Absolutism and universalisation are often based on perceived inalienable facts of nature (Aristotle, C4thBCE/2004),^71^ such as gender relations, and racial hierarchies, which necessitate certain behaviour. FE challenges these so‐called ‘Natural Laws’, viewing them as socially constructed to maintain systems providing androcentric and colonial power and privilege.^98^ For this reason, we should ‘question everything that claims to be universal,^98^ ^,p.19^ drawing our attention to ‘unquestioned answers’^99^ which often reinforce subjugation and oppression by being rooted in the ideals of rational (white) man.

#### 3.1.2 Rationality and embodiment

Enlightenment ethics are contentious as they are hyperrational^29^ and represent elitism through the ideal man.^28^, ^29^, ^98^ In this androcentric ideal, women are often excluded from the possibility of rational thought and are seen as being unable to reach the same stage of moral development as men^67^ due to their emotionalism.^28^, ^98^ Similar exclusions to rational thought have historically been applied to non‐White individuals,^76^ with much enlightenment thought failing to even consider non‐White men or women in their discussions. Although it is possible to argue that Kant valued all lives as demonstrated by his gender‐neutral terminology,^100^, ^101^ others see his ethics as fundamentally racist.^102^, ^103^ Ultimately his writings are steeped in historic biases which limit the potential of non‐White, nonmale individuals.^28^, ^100^ In this archetype of power and privilege^101^ those who do not conform (women, children, people of colour, the physically and mentally impaired) are marginalised.^28^ Feminists argue that we must examine our professional lives to ensure we are not perpetuating this structure of oppression. FE wants to reclaim rationality that embraces emotion, experience and intuition (embodiment) in moral decision‐making.^100^, ^104^, ^105^ For the SVRS, this raises many questions, including what educational models we ascribe to and what is the impact of these on our clients? How is experience‐based learning and personalisation valued? What if practitioner and/or client intuition contradicts theory? Our understandings of embodiment in the context of voice are largely based on the white European male model of voice^106^ and therefore, our approach to embodied knowledge in voice pedagogy and rehabilitation requires divorcing, or at least expanding from the normative white male archetype of voice science.

The feminist reformulation of Virtue Ethics as *Character Ethics*,^107^, ^108^ has tried to assist. It does not rely on universal principles, so could be socially constructed, providing a more person‐centred and nuanced framework, which could underpin personalised and experiential models of vocal pedagogy. Dillon^107^ argues that what is needed is not an abandonment of traditional virtues, but a removal of their genderisation and recognition of the interconnectedness of private and public values and action.^107^ However, the question remains: who decides what desirable characteristics are? Metaethical issues such as ‘what is good’ are important to examine in any ethical debate. We must critically evaluate who is deciding the moral standards by which we live and hold one another accountable. Ethical relativism may be congruent with feminist principles of subjectivism and relativity and would also account for alterity in conceptions of caring practices, such as may be found in approaches to singing voice rehabilitation across the globe. However, questions are raised concerning whether actions permitted in one context would be permitted in another. If the answer to such examination is no, then we are forced to ask whether the original ‘good’ action really was justifiably so. Intersectionally‐minded critics of Hobbes, Kant and Mill draw attention to the racist anthropological perspectives in their writings^103^ that for many form the basis of understandings of such value terms. This is an important criticism of feminist character ethics; who has the power to determine the moral worth of another person's values? For those involved in decolonising projects, these character judgements are an important ethical and social legacy, which need to be challenged in our ethical maxims and prescriptions.^76^

Whilst not immune from intersectional feminist criticism, Aristotle's virtuous mean (where a virtuous person is one who operates in the space between the extremes of excess and deficiency) may provide an answer to this criticism, as it positions ethical behaviour on a spectrum, allowing for a degree of flexibility and potentially relativism. However there are still some concerns over subjectivism and relativism here, as how we define the terms used on that continuum from virtue to excess may also differ across cultural and historical contexts.

#### 3.1.3 Power and vulnerability

If we understand vulnerability as ‘susceptibility to harm’,^28^ ^,p.201^ then the bio‐ethical maxim of ‘do no harm’ must remain central for SVRS. However, FE argues this maxim is problematic as being in a position to ‘do no harm’ necessarily presumes a position of power over another. It is this hierarchical view of care, education and leadership which may contribute to moral dilemmas. For example, consider SVRS epistemology: what knowledge do we value—expert—knowledge or self‐knowledge? So often, practice assumes expert knowledge is supreme,^37^, ^38^, ^39^ with white western knowledge being hailed as the gold standard in health, well‐being and social contexts.^48^, ^76^, ^95^ We must be careful that SVRS moral decision‐making does not make this assumption. Nor should we set these two knowledges in opposition; our ethics should seek to synthesise them in practice.

Adopting a feminist ethic of care may ameliorate this issue. Care is about resisting hierarchies and power structures as it considers how we are of equal value and worth by virtue of being human.^109^ Vulnerability becomes the foundation of the approach,^110^ however, vulnerability remains entwined with power and responsibility, and responsibility is inextricably linked with legalism which upholds the absolute and idealistic.^98^ Despite advocates of intersectional feminist care ethics trying to resist this by contextualising moral decision‐making and critiquing the concept of alterity in care,^95^ critics argue context *is* the problem, as care is often conceptualised from its archetype in the private sphere of mothering. Such conceptions can reinforce female stereotypes,^111^ leaving women open to exploitation. The conflation of care and femaleness does not account for different experiences and definitions of female, neither accounting for cultural and geographical differences such as race, nor acknowledging the variance of gender and sexual identity. What about those assigned female at birth who no longer identify as female? Are we to say that by category definition they do not have caring attributes, or that they are more caring than those assigned male at birth? For most, an intersectional feminist analysis is not needed to eschew this claim.

Furthermore, stereotypical definitions of care assume that care as a feminine virtue is not a quality or practice that can involve men, which not only limits men's potential full self‐expression contributing to a toxic machismo which is as harmful to men as to women,^112^ but also contributes to the propagation of professional hierarchies in our health care institutions (where (straight white) men are more often in higher paid managerial positions rather than front‐line caring roles.^113^

Further practical complications for care ethics are added by FE insisting on the importance of *self‐*care.^114^ When should SVRSs prioritise their own needs as opposed to their clients? How far should these considerations be based on the impact on the clients; when does selfless become selfish and vice versa? Traditionally, even enlightenment ethics has been heavily influenced by Christian antecedents, with the image of Christ‐like self‐sacrifice being held as the highest virtue.^29^ This ensures continuance of public and private structures which limit and restrict equality of opportunity, as diminishing the importance of self‐care enables devaluing of caring activities.^114^ Ought *self‐*care proceed conceptions of caring responsibility to others? Should self‐care be viewed as ‘a foundation for freedom’,^114^ ^,p.21^ and a radical restructuring of traditional notions of autonomous agency.

#### 3.1.4 Autonomy and interconnectedness

Care ethics is collaborative problem solving.^115^ However, TE decision‐making is individualist and predicated on autonomy. Autonomy is challenging for feminists as ‘relations matter in structuring society’.^116^ ^,p.66^ We do not live in isolation but within ‘webs of relationship’,^27^ ^,p.92^ ^0^ and everything we think or do is influenced by our experiences, including how we experience others. A problem with patriarchal enlightenment structures is that the focus on autonomy serves to ‘divide the self and separate[s] it from others’.^109^ ^,p.1^ ^3^ This again demarcates the separation of private from public leading to places in society that are hidden from ethical scrutiny and open to abuse.^117^

With regard to interconnectedness, one may decide that Utilitarianism recognises morality as socially connected, however, it requires impartiality, which can lead to unethical action. For example, SVRS may try a new strategy with a client and it may harm them. However, as only one person has been affected, a Utilitarian may not see it as immoral if my learning stops others from using it again. It is in view of this that some FE scholars reframe autonomy.^100^ Scholars such as Dillon^107^ emphasise the importance of free will and resisting coercive practice. For the SVRS, such a focus may lead to awareness of potentially harmful power dynamics between practitioners and questioning whether clients have ownership over their rehabilitation. On the other hand, the value this places on independence over inter*dependence*,^28^ can lead to non‐inclusive practice. As social beings, morality is influenced by the context in which we live,^98^ requiring communities of practice. Within these communities SVRS may need to relinquish positions of power and influence.^110^

### 3.2 Implications of ethical theory for SVRS policy

If the DA finds that SVRS policy is founded on intersectional FE then this would support practice that is caring and equitable. It would encourage SVRS to put clients' needs, perspectives and experiences at the heart of their work *alongside* attention to their own self‐care. It would value input from all parties involved in the rehabilitation of voices, not simply those deemed experts. This would empower clients' autonomy, potentially leading to improved outcomes through greater engagement with strategies that have been selected mindfully in consideration of an array of contextual features.

However, if the antecedents of policy are rooted in TE, then this would situate it directly at odds with current moves towards patient‐centred, BPS approaches.^30^, ^31^ In TE, the balance of power is shifted in favour of (often white heterosexual male) experts, which may not only disenfranchise clients, but may also exclude SVRS practitioners (who are mainly women) from using their skills and expertise as part of a multidisciplinary team. Such context would contribute towards dissonance, rather than relationship within professional networks and may make it harder for the SVRS to safely navigate the boundaries of their combined pedagogical and health care knowledge and skills.

## 4 DA FINDINGS

### 4.1 What are the ethical antecedents of current SVRS ethical policy?

The first finding to acknowledge is that each document has been produced in the global north. It is important to recognise that, whilst the personal identities of all who contributed to these policies was not available, the geographical location is significant for an intersectional analysis and suggests that we consider the Western and Eurocentric values, experiences and (un)conscious biases which may underpin their content.

**Figure 3:**
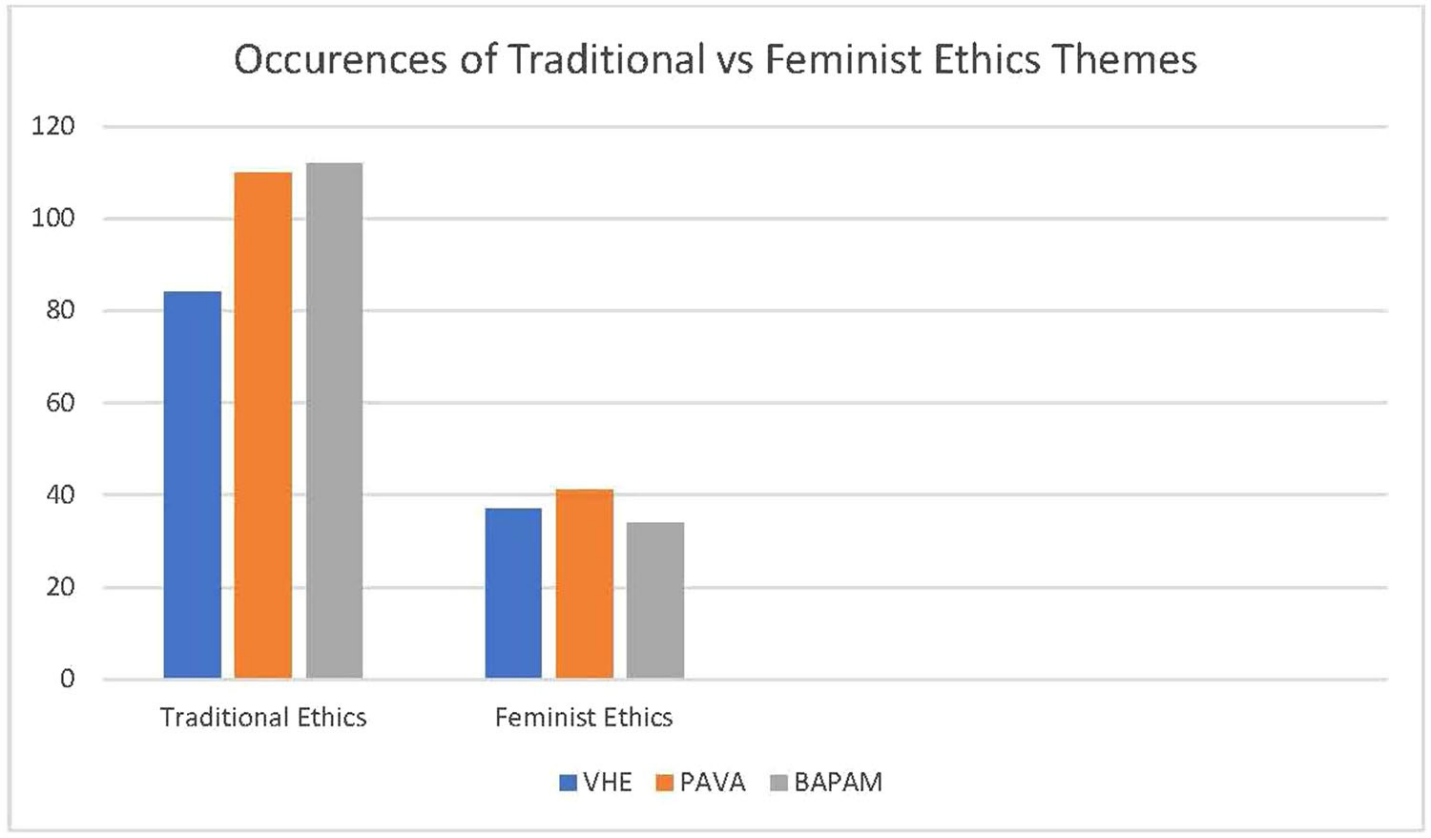
Occurrences of traditional and feminist ethics in reviewed documents.

**Figure 4:**
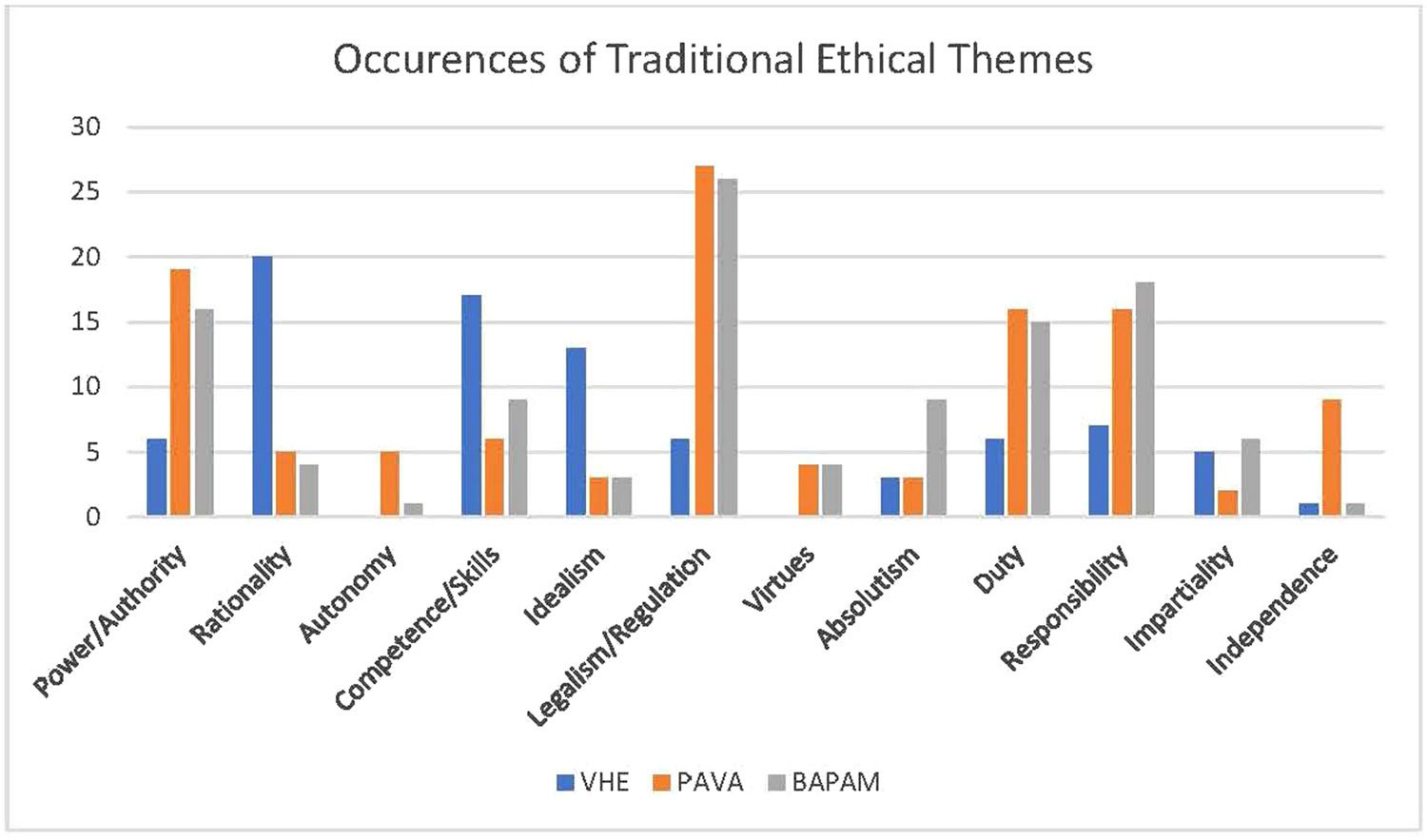
DA findings—themes in traditional ethics. DA, document analysis.

Perhaps as a consequence of this geographical context, DA found that each policy was heavily weighted towards traditional ethical theory (Figure 3). Excepting virtue and autonomy, each theme extracted from open coding of TE was present, with considerable evidence of the enlightenment androcentric themes of power, rationality and legalism (Figure 4).

For example, in the VHE document, rationality was evidenced via the focus on analytical competencies such as the requirement for advanced technical training:

‘The Voice Rehabilitation Specialist (VRS) will have had further training in:

- Endoscopic evaluation of laryngeal function and observation of a range of pathologies
- Anatomy—a day in the dissection lab
- Advanced functional anatomy of the healthy voice’.

*(VHE, 2022)* ^53^

In the BAPAM document power was evidenced through requirements for externally assessed training and providing documentary evidence of observational experience validated by professionals in positions of authority within the medical profession:

‘1. Hold or have previously held a contract with an NHS specialist voice clinic…verified by contract documentation.

2. […]

3. Work under clinical supervision…letters from clinical supervisors confirming monthly sessions are required.

4. […]

5. […]

6. Have completed endoscopic interpretation…’

(BAPAM, 2017/2022).^51^, ^118^

For PAVA, the legalistic focus was evidenced throughout the document with frequent reminders to ensure compliance with local and national laws:

‘d. PAVA recognises there are local, state, region, province, and/or national licensure regulations which govern who may work with persons with communication disorders and how that work may be performed. It is the responsibility of each member to ensure that his or her work with persons with voice concerns does not violate the scope of practice set for by the regulations in the location in which the work occurs’.^119^

**Figure 5:**
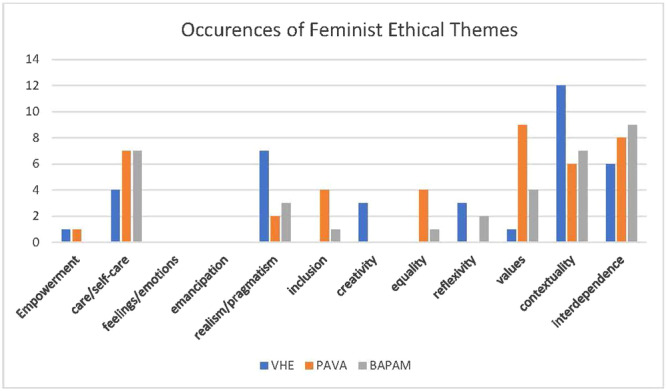
FE themes. FE, feminist ethics.

Across all documents there were far fewer occurrences of feminist ethical themes (Figure 5).

Of note is the absence of emancipation and embodiment. This is particularly interesting for VHE as for an organisation that vociferously advocates BPS practice, there seems to be a disconnect between policy and practice. Likewise, lack of empowerment in BAPAM's policy appears at odds with the organisation's mission to ‘connect[s] those working and studying in the performing arts with clinical specialists…’ for the care of individuals' well‐being and the advancement of their career aspirations.^118^ This mission would be further advanced through the addition of care ethics to develop the current legalistic and competency‐based policy into a more person‐centred vision. Again, this finding highlights policy/practice inconsistencies when viewed through the feminist lens.

As evidenced in the above quotation from BAPAM's mission statement, across all documents there were some links to FE themes, notably interconnectedness and contextuality. These were evident in discussion of client/practitioner relationships, as well as recognition of the importance of respectful communication between networks of professionals:

‘Will often be working within a multidisciplinary voice clinic’.

‘Know and understand the roles of professionals who are part of the rehabilitative voice team’.

‘Know when to refer singers’.

(VHE, 2022)^53^

‘…communicate clearly with colleagues…’

‘…seek regular advice from performers that you see from a BAPAM clinical supervisor…’

(BAPAM, 2017/2022)^51^, ^118^

‘1a…recognise and encourage the right of the patient, client or student to participate in the treatment or habilitation process.

3b…consultation and referral must be sought where appropriate. Communication with colleagues must be truthful and forthright’.^119^

However, generally FE themes are sparse and where found were largely espoused through clauses relating to legislative contexts and the dependence of professionals on each other for maintaining the reputation of the profession as a whole. In this context these themes then became more closely aligned them with TE:

‘3d. PAVA members shall uphold the dignity and autonomy of the various voice professions…

3e. PAVA members shall not participate in any form of conduct that adversely reflects on the voice professions’.^119^

This is not necessarily problematic, as FE can seek integration with elements of TE where that provides credibility.^120^ However, in the absence of other aspects of FE, such as inclusion, embodiment, reflexivity and care, the contents of these documents could be developed further to provide an ethical framework that can serve the best interests of both clients and practitioners within a holistic BPS context.

## 5 DISCUSSION

### 5.1 What are the implications of current SVRS ethical policy documents on SVRS practice when viewed through the pragmatic feminist poststructural lens?

#### 5.1.1 Absolutism and relativism

Each document was weighted towards an absolutist approach, grounded in themes of duty,^68^ responsibility to the majority—professional reputation^65^, ^66^ and legalism.^70^ In particular, PAVA's focus on legal compliance is out of alignment with FE as it only partially considers the relational aspects of practice and the socially constructed nature of ethical action. Whilst one must obviously work within the law, this needs to be balanced with the needs of individual cases.^121^, ^122^ A traditional legalist approach may in fact cast doubt over whether singing teachers should be engaging in rehabilitation at all, as it can be argued that it is outside of their scope of practice. A feminist care approach might suggest this would be detrimental to clients and, where singing teachers have skills supported by appropriate training, it might be in clients' best interests for them to be used.^31^ Applying intersectional feminist post‐structural questions to this issue suggests that the ethical underpinning of SVRS policy may in fact be resting heavily on patriarchal structures that are protectionist and serve to restrict access to the profession of rehabilitation through knowledge gatekeeping.^123^, ^124^, ^125^ Although presented as policy that safeguards clients, when deconstructed, the underlying concepts could be interpreted as self‐serving to those who wish to protect their status within the hierarchy of caregiving.

This protectionist stance is also evidenced through clauses which describe practitioners' duty to maintaining standards of ethics so as not to bring the profession into disrepute. Explicit concern for public perception and professional reputation, as highlighted in the BAPAM and PAVA examples above further aligns them with absolutist Utilitarian principles.^65^, ^66^, ^126^, ^127^ Concern for how the action of individual practitioners may impact the greater number (the profession) presents an egoist self‐interest that challenges feminist principles. These clauses prioritise the public face of ethics over the private, which may have consequences for ethical practice within the private studio. FE criticises TE for this focus on the public, arguing that it leaves room for abuses within private spaces.^114^ Much SVRS work is in private studios, where there is little opportunity for public accountability. Coupled with the lack of a specific SVRS legislative body and whistleblowing procedures, this leaves all parties vulnerable to abuse. Although national safeguarding organisations provide help and support for victims of major abuses, lack of provision supporting private accountability structures in SVRS practice means that less insidious injustices – such as unconscious bias—may go undetected.

The absence of supporting resources in these documents is part of the problem, as they do not help practitioners to negotiate contextually individual subjective situations Feminist poststructuralism suggests this is evidence of tacit acceptance of dominant patriarchal structures, with lack of consideration for contexts outside of social norms leading to dubious ethical practice by omission and a lack of critical intersectional thinking. Intersectional feminism demands that policy ought to explicitly focus attention on marginalised contexts to ensure that discriminatory and unfair practices are avoided. The VHE scope of practice document does imply that contextuality has been considered by virtue of the language employed in some clauses, for example, ‘broad ranges’, ‘minimums’, ‘may have’ and ‘will often’, however it could go further by specifically addressing the range of contexts within which the SVRS may face ethical conflicts. VHE does provide critical thinking and inclusivity training, however this analysis has highlighted incongruence between their policy and practice.

BAPAM and PAVA policy also begin to address relativity and contextuality in references to anti‐discriminatory practice and practitioners' attendance to the laws of their state:

‘2b. PAVA members shall not alter their delivery of services and/or training on the basis of race, ethnicity, gender, gender identity, gender expression, age, religion, national origin, sexual orientation, deficit, disability, cultural background or socioeconomic status, unless such alteration of delivery of services or training is necessary to best meet the special needs of unique populations’.^119^

‘…you must not discriminate against individuals or let your personal views affect your professional relationships or advice, and you should challenge discrimination if it comes to your attention’.

(BAPAM, 2017/2022)^51^, ^118^

However, the discrimination guidance is disproportionately small when considered within the entire policies. There is either an assumption of ethical knowledge and shared values between organisations and practitioners, or it has not been considered. This is problematic for the SVRS context, as without accredited and regulated training and supervision such knowledge of theory and how to apply it cannot be assumed. At present, anyone can call themselves a vocal health expert or rehabilitation specialist regardless of level of training and experience, and therefore, further development of these documents to include explicit guidance on areas such as anti‐discriminatory practice would be beneficial to ensuring safe, effective and professional practice.

#### 5.1.2 Autonomy and interconnectedness

A further issue of inclusivity is present, as the traditional ethical structures within these documents privilege expert knowledge, which can detract from a client‐centred focus. Intersectional feminist analysis of this would lead to questioning of how far clients' autonomous participation in rehabilitation is valued by these organisations. The VHE document in particular could be developed in line with VHE values by the inclusion of information that is explicitly client focused. Implicitly, all its present contents contribute to practice that means SVRSs can meet the holistic needs of clients, however the addition of statements that address relational behaviours, expectations and safeguards would strengthen their policy in line with their BPS values, bringing policy and practice into congruence.

BAPAM and PAVA go a little further in respect of safeguarding, with each referring to insurances and BAPAM referencing Disclosure and Barring Service checks. BAPAM also refers the SVRS to its ethical standards document for educators, within which lies a more explicit client‐centred focus:

‘1. Promote and protect the interests of individual performer patients.

As a provider of care and advice, you must treat individual performers with respect, listen to their views and involve them in decisions about their own health and well‐being, including providing information and seeking consent to the actions you propose…’^118^

PAVA has a similar clause where it encourages all members to ‘recognise and encourage the right of the patient, client or student to participate in the treatment or habilitation process.’^119^ ^,p.1^ The development of material resources to accompany the symbolic framing of autonomy within these policies would strengthen these documents as practitioner resources. Providing exemplary material on how such principles can be applied in practice, for example considering the practical realities of what should happen when there are conflicts of interest between clients and practitioners, would develop these documents from regulatory texts into emancipatory materials. Thinking through specific examples is concerned better for intersectional feminist practice, as they centre the person and the subjectivity and help to encourage critical reflexivity in practitioners rather than a one size fits all approach based on some objective notion of virtuous action.^128^ Again, in the absence of formal SVRS training it is suggested that too much theoretical and practical ethical knowledge has been assumed. Until more robust training and accountability structures exist for the SVRS, developing these policies along feminist lines would be an excellent and pragmatic means of ensuring clients have autonomy in their rehabilitation processes.

#### 5.1.3 Power and vulnerability

In accordance with TE principles, practitioners are positioned as authority figures in these documents, through requirements for empirically rational training that centrally position practitioner knowledge within the rehabilitation process. Although advanced and accurate knowledge is crucial for safe practice, these hierarchies are problematic for intersectional feminism as they privilege hyperrational approaches over embodied epistemology. Embodiment is centrally important for singing^129^, ^130^, ^131^ and this needs further acknowledgement and development in the policies. Without recognition of the centrality of embodiment there remains a danger that client perception is devalued as their experiential knowledge is reduced to secondary importance. When examining the mission statements of each organisation it is clear that in this respect their policies do not reflect their values, which are client centred. To develop greater congruence between policy, mission statements and practice, feminist care ethics could be employed to develop and expand upon current contents. This might include ideologies of co‐constructed rehabilitation practices, and links to resources supporting the development of practitioner counselling skills (with guidelines that indicate clearly where boundaries lie between this and psychotherapy and how practitioners can avoid overstepping them).

As well as these suggestions from care ethics, it is also noteworthy that none of the documents recognised this position of power in a way that aligned with current ethical guidelines in western medical practice. For example, the exhortation to ‘do no harm’^132^ is not found in any document yet is a central tenet of health care practice. Whilst omissions like this may be the result of assumptions, developing its explicit inclusion in these policies would be significant in upholding standards and safeguarding. From the perspective of pragmatic feminist post‐structuralism greater clarity of foundational values is vitally important for ensuring the creative power of texts aligns with organisational aims in policy and practice.^27^

Further to this, it is important in FE to consider the implications of current policy and practice on practitioners and *their* vulnerabilities. Part of feminist care ethics is the importance of self‐care. Both the PAVA and BAPAM documents refer to practitioners' well‐being:

‘5. You must make changes to how you practice or stop practising if your physical or mental health might affect your judgement or ability to carry out your practice or put others at risk for any reason’.^118^

‘2 g. In professional settings PAVA members shall maintain good physical and mental health and self‐care in order not to jeopardise the health, well‐being and safety of others’.^119^

Although self‐care is present, in each instance this is in relation to responsibilities towards clients. For FE this does not represent true self‐care, as it still embeds a hierarchy by prioritising the well‐being of clients over practitioners.^114^ Developing this aspect of feminist care, without a reliance on traditional notions of duty to others would provide further emancipatory structures for professional practice.

#### 5.1.4 Rationality and embodiment

Intricately connected to ethics of care and self‐care, is the theme of embodiment. Each of the documents analysed could further develop along emancipatory themes by supporting practitioners to trust their own embodied knowledge of their needs. At present, practitioner self‐care is rationalised along utilitarian lines, which consider how, by addressing their own needs, practitioners may impact on the well‐being of clients.^65^, ^66^ There is a strong element of Kantian duty within this,^68^ that may prevent practitioners from feeling able to exercise effective self‐care. Moving from addressing practitioners as sources of rational authority, towards skilled, caring, embodied practitioners would not just provide symbolic affirmations of self‐care, but could lead to greater outcomes for patients, as well‐being research highlights that when practitioners are well‐resourced through genuine self‐care they are better able to meet the holistic needs of clients^133^ through practice that creates a safe space for the achievement of relational depth.^9^, ^19^, ^134^, ^135^ In consideration of care ethics, the potential for conflicts of interest between client‐centred care and practitioner self‐care also need addressing, for example when a practitioner needs to command a certain fee but a client cannot afford this—whose needs are prioritised? Again, in line with best practice recommendations,^89^, ^90^ supporting resources must be provided alongside policy as one cannot assume practitioners have the knowledge and skills to address these issues.

Further consideration of embodiment is warranted in relation to training and supervision. BAPAM calls for practitioners to undergo supervision where SVRS can discuss ethical issues and seek clarity through consideration of alternative perspectives. However, strong orientation towards rational and logical skills suggests that embodied knowledge is valued less highly than knowledge gained through rational empiricism. As discussed above, this has implications for how clients' perspectives are valued and it also has consequences for the rehabilitation methods adopted by practitioners. Such a hyperrational focus may lead to the view that some methods are unethical, when in fact they are simply countercultural or unorthodox. Whilst there clearly remains a place for evidence‐based practice in SVRS, intersectional FE demands that this is not privileged over other forms of knowledge and practice. We must consider that there is power, truth and benefit in forms of knowledge other than those traditionally privileged in western Eurocentric thought.^76^ Embodied, socially constructed methods, although presently out of vogue in western structuralism should not be viewed as synonymous with unethical practice.

## 6 CONCLUSION

### 6.1 How might a feminist paradigm contribute to the future development of SVRS ethical policy and practice?

Adopting the guiding principles of intersectional FE for singing voice rehabilitation would present a radical shift in policy, but perhaps a closer alignment to existing practice. This study proposes that embracing an ethic of relational, embodied care and intersectional justice would contribute to the creation of policies that are better able to address the holistic needs of clients and practitioners. Central to this is recognition that rational autonomy is not a marker of the ideal human, but one damaged by patriarchal structures; one who is not experiencing the full range of experience and perception.^117^ True human function is relational and emotional and when we deny these aspects in moral decision‐making, we are limiting our potential, often to preserve structures that perpetuate power and privilege.^99^ If current SVRS policies are to achieve congruence with the practical aims, values and mission statements of their organisations, then they require development to ensure they are emancipatory texts that support the full flourishing of human potential. Their current textual alignment with rational, universal autonomy risks diminishing the importance of embodiment, relationship and context in rehabilitation practice. Part of the problem is the lack of acknowledgement that SVRSs hold a legitimate role in voice rehabilitation as part of a multi‐discipline team. This is already being addressed in some areas of vocal health through multi‐disciplinary approaches. Pragmatic feminist ideology may help address the legitimacy issue further by encouraging a move away from patriarchal power structures and knowledge gatekeeping, towards more inclusive and relational professional networks and communities of practice.

Within these communities, policy needs to be developed in such a way that it facilitates critical and reflective ethical thinking that embraces intuition, emotion and logic. In Clark‐Miller's^28^ critique of Kantian rationalism, she looks towards an ethic of care as a way of restoring moral values based more fully and realistically on the human condition. Whilst this is not a perfect way of being ethical it is one that ought to be considered strongly for professions such as mine which nurture the health and well‐being of others. Values of love and relational depth^135^ are important for SVRSs, and represent a move away from public facing, legalistic ethics towards moral behaviour that also considers the values of private, inclusive practice. Character ethics adds an interesting dimension to this as it considers the values to which SVRSs might subscribe. When one compares SVRS policy with other examples of best practice, such as the guidelines from BACAP and the ICF, it is evident that formulating statements of mission (intention) and character (values) would transform these documents into resources that could empower SVRS practice. Inclusion of these in policy would help to ensure practitioners are equipped to work in a way that facilitates client autonomy. Additionally, their presence could provide the means for a regulatory body to uphold standards and take appropriate action where they are deemed to have not been met. Explicit inclusion of these value statements in policy supports the evolution of policy from a symbol of intent towards a practical and affective document for change.

Whilst current documents already go some way to facilitating care through consideration of safeguarding legislation, to accurately reflect the complexities of practice, policy must provide clearer material as well as symbolic guidance beyond a ‘thou must not’ approach. Policymakers must be aware that ‘translating … principles into any concrete application inevitably means adapting to the local, historical, socio‐political and economic conditions’,^136^ ^,p.154^ and that policy must be interactive rather than legislative,^98^ acknowledging the performativity of text. To facilitate this, best practice in feminist policymaking recommends including exemplar material that illustrates clearly how the symbolic and ideological facets of policy can be enacted in practice.^128^ In creating these resources, policymakers need to address the challenges posed by the socially constructed nature of transdisciplinary BPS practice. As well as providing practitioner training, this requires policymakers to confront their own unconscious biases (and those of the systems within which they operate). At present there are no specific resources to support voice pedagogy policymakers to achieve these aims. Therefore, before the contents of documents can be developed, a first step towards producing feminist SVRS policy and practice is to further support the development of feminist policymakers. This requires an educative framework promoting critical evaluation of subjective contexts through the resourcing of reflective practice in a network of ethical practitioners. Developing a feminist policy through such a community of practice may go some way to providing a resolution to the wider ethical debate of how voice rehabilitation by specialist singing teachers can be ethically practised.

## CONFLICT OF INTEREST STATEMENT

The author declares no conflict of interest.

## Data Availability

Research data are not shared.
